# The Nature of Consumption

**Published:** 1883-02

**Authors:** 


### THE NATURE OF CONSUMPTION.

If a green bush is pulled up by the roots, and these roots are cut off close to the body of the bush, a good general idea may be had of the "*Air Passages*," if this bush is turned upside down. The end of the bush next the ground represents the part of the throat where the voice organs are, the body of the bush represents the windpipe, the branches of the bush represent the bronchial tubes, the leaves of the bush represent the lungs themselves: and as the leaves cover the branches from sight, so the lungs, which are nothing more than little bladders distended with air, hide the bronchial tubes. Here then are four distinct parts of a great apparatus, each different in locality, and each locality the subject of a distinct disease, requiring different remedies and a different treatment. Liquid guano destroys the leaf, but gives life to the roots. Water does but little good if thrown over a tree, but saves it from dying if thrown on the ground about it. So what would benefit one part of the air-passages, might be wholly unavailing if applied to another part. What would cure a disease of the windpipe, might destroy the lungs, or be perfectly useless. Thus showing how important it is to know certainly what the disease is, and where it is located, in reference to the great fountain of life. the breathing apparatus.

